# Association between insulin resistance and abnormal menstrual cycle in Chinese patients with polycystic ovary syndrome

**DOI:** 10.1186/s13048-023-01122-4

**Published:** 2023-02-23

**Authors:** Jiali Niu, Meiyin Lu, Bin Liu

**Affiliations:** Department of Biobank, Shenzhen Baoan Women’s and Children’s Hospital, Jinan University, Shenzhen, 518102 Guangdong People’s Republic of China

**Keywords:** Polycystic ovary syndrome, Insulin resistance, Amenorrhea, Risk factors, Sex hormone

## Abstract

**Background:**

Polycystic ovary syndrome (PCOS) is a common reproductive endocrine disorder, which is characterized by insulin resistance (IR) and menstrual cycle disorders. IR is thought of as a pivotal cause of PCOS and related comorbidities. However, the link between IR and abnormal menstrual cycles in PCOS should be further studied. In this study, we clarified the dose–response relationship between IR and abnormal menstrual cycles in patients with PCOS.

**Results:**

In this retrospective study including 140 patients with PCOS, we found that there was a dose–response relationship between the increased HOMA-IR index and the level of menstrual cycle disorders (1.61 [95%CI: 1.37–1.85] for normal menstruation, 2.02 [95%CI: 1.61–2.44] for oligomenorrhea, 2.35 [95%CI:1.96–2.75] for amenorrhea, *P* for trend = 0.003). Further stratification analyses showed that this dose–response relationship was more evident in the patients who were younger, had higher BMI, higher AFC numbers, elevated levels of testosterone, anti-Müllerian hormone, inhibin B, and prolactin levels, and had a lower progestogen level.

**Conclusions:**

Our study has established an association between IR and abnormal menstrual cycles in patients with PCOS, which can be affected by age, BMI, and hormone levels. Our results might be helpful for further prevention and treatment of amenorrhea in PCOS.

**Supplementary Information:**

The online version contains supplementary material available at 10.1186/s13048-023-01122-4.

## Background

Polycystic ovary syndrome (PCOS) is a common reproductiveendocrine disorder in women of reproductive age today [1, 2]. Studies have shown that its current prevalence is 5.5%-16.0% [3]. PCOS is characterized by polycystic ovaries, oligomenorrhea or amenorrhea, hyperandrogenemia, insulin resistance (IR), weight gain and infertility [4–7]. It can lead to various health problems, such as cardiovascular disease, diabetes and hypertension [8]. The cause of PCOS is not clearly stated, but many signs show a strong link between the metabolic and endocrine system.

Clinical manifestations of menstrual cycle disorders include oligomenorrhea and amenorrhea. Some studies have shown that about 30% of patients with PCOS exhibit normal menstruation, 85%-90% of women with scanty menstruation have PCOS, and 30%-40% of women with amenorrhea have PCOS. The current diagnostic criterion for amenorrhea in patients with PCOS is a period of menolipsis for more than three months, but many patients with amenorrhea did not pay attention to this symptom and were not timely diagnosed and treated. Additionally, miscarriages and adverse pregnancy outcomes are usually seen in patients with PCOS, particularly in those with amenorrhea [9]. Therefore, early diagnosis and intervention for amenorrhea in patients with PCOS are essential.

IR and menstrual cycle disorders are characteristics of PCOS. When a woman's hormone levels are affected, this in turn disrupts ovarian function, which lead to menstrual complications such as anovulation and amenorrhea [10]. Currently, in addition to menstrual cycle disorders, IR in patients with PCOS has a significant impact on the health of women of childbearing age, which is measured by the homeostasis model assessment: insulin resistance (HOMA-IR) index [11, 12]. As sex hormone secretion influences the menstrual cycle, IR interacts with the patient's body estrogen levels, higher estrogen levels can increase luteinizing hormone (LH) secretion and decrease follicle stimulating hormone (FSH) secretion, which in turn leads to follicular membrane cell and granulosa cell hyperplasia. In patients with PCOS, insulin increases the sensitivity of the adrenal cortex to the activationof adrenocorticotropic hormones, which further increases androgen secretion and consequent disruption of the menstrual cycle [10, 13]. In addition to sex hormones, both Anti-Müllerian hormone (AMH) and inhibin B (INHB) are secreted by ovarian granulosa cells, and both are affected when patients experience disruptions in their menstrual cycle. Some studies have shown that AMH and INHB are negatively correlated with HOMA-IR [14, 15].

IR is a key characteristic of PCOS and is associated with worsening of other PCOS features, it increases the risk of infertility and cardiovascular disease in the patients with PCOS [4–8]. As for abnormal menstrual cycles, a study published in 1993 reported that it is significantly associated with IR [16]. From then, some studies have studied this association between IR and the menstrual cycle in PCOS [17–20]. However, there were some limitations of the study design in these studies, or they did not well control the important counfounders/biases to assess the association between these two indexes in PCOS. As for the study design, some studies excluded the PCOS patients with oligomenorrhea [16]; while others combined the oligomenorrheic/amenorrheic patients into one group without comparing them with each other [17, 18]. The reported associations between IR and menstrual dysfunction were incomplete in these studies. As for the counfounders/biases, some studies did not consider the role of hormones such as testosterone [19]; while others did not use multivariate analysis models to compare the HOMA-IR levels among the PCOS with eumenorrhea, oligomenorrhea and amenorrhea [20]. Hence they did not report a significant increasing trend of HOMA-IR scores in these patients. Therefore, this study was conducted to further clarify the correlation between IR and menstrual cycle abnormalities in the patients with PCOS. Because the dose–response relationship is a powerful instructor for quantitatively showing the strength of association [21–23], and is widely used for metabolic indices [24, 25], we used the linear trend model in Multivariate Analysis of Variance (MANOVA) to assess the dose–response relationship between IR and abnormal menstrual cycles in PCOS.

This study aimed to illustrate the dose–response relationship between IR and menstrual cycle abnormalities in patients with PCOS in a Southern Chinese population, and to provide a theoretical basis for early diagnosis and treatment of amenorrheic patients.

## Materials and methods

### Ethical considerations

This study was conducted according to The Code of Ethics of the World Medical Association (Declaration of Helsinki) and was approved by the Ethics Committee of Shenzhen Baoan Mothers’ and Children’s Hospital, Jinan University. The approval document serial number is LLSC-2021–04-02–04. The study was conducted with the informed consent of the subjects to collect their clinical information.

### Study population

This retrospective study included 140 patients with PCOS who were aged 20–40 years, and were newly diagnosed with ultrasound examination between August 2019 and December 2021 at Shenzhen Baoan Mothers’ and Children’s Hospital, Jinan University. The patients were followed until April 2022 to confirm the diagnosis of oligomenorrhea and amenorrhea.

### Exclusion and inclusion criteria

Exclusion criteria for patients with PCOS: patients with other infertility disorders such as combined uterine fibroid, endometriosis, and tubal lesions; patients taking oral contraceptives for birth control; in addition, patients with adolescent PCOS were excluded from this study because the diagnostic criteria for adolescent PCOS are still very controversial at home and abroad.

#### Acquisition of parameters

Clinical parameters of these patients were collected for each subject from the Hospital Information System (HIS) and Laboratory Information System (LIS) of our hospital, including age, number of pregnancies and deliveries, antral follicle count (AFC) and clinical presentation. Additionally, a series of fasting biochemical parameters were also screened, including blood glucose and insulin. Progesterone, testosterone, FSH, LH, estrogen and prolactin were measured on days 3–5 of the patient's menstrual cycle. In addition to the sex hormones, we tested blood specimens from the subjects for AMH and INHB. We used HOMA-IR, quantitative insulin sensitivity check index (QUICKI), and insulin sensitivity index (ISI) are used to assess the level of IR [26, 27]. As HOMA-IR is currently used more often and has a better response to the level of IR, this study mainly uses the HOMA-IR indicator for assessment. Meanwhile, other two indexes were also analyzed. As for menstrual status, the patients with PCOS in this study were divided to normal menstruation, oligomenorrhea, and amenorrhea group, respectively. The HOMA-IR index was calculated as (insulin [mu/ml] × glucose [mmol/l]) / 22.5 [13]. QUICKI index was calculated according to the formula: QUICKI = 1/(lg (I0) + lg (G0)), where I0 denotes fasting insulin and G0 denotes fasting glucose27. Insulin sensitivity indices for glycemia [ISI (gly)] can be calculated using the formula: ISI (gly) = 2/[(INSp*GLYp) + 1], where INSp, GLYp = insulinemic and glycemic areas during OGTT (75-g glucose) of the person under study, this study by considering data at 0, 1, and 2 h (0–1–2 h areas). Expressed as unit/volume · h^−1^, 0–1–2 h area is equal to 1/2 value at 0 min + value at 1 h + 1/2 value at 2 h. Instead of areas, basal levels can also be used. Basal levels and areas are expressed taking the mean normal value as unit [28]. The laboratory is accredited in accordance with ISO 15189:2012 Medical Laboratories-Requirements for Quality and Competence (CNAS-CL02 Accreditation Criteria for the Quality and Competence of Medical Laboratories) for the competence to undertake testing service as described in the study. We determined sex hormone, glucose and insulin levels using ARCHITECT i2000 Microparticle Chemiluminescent Immunoassay Analyzer (Abbott Laboratories, Abbott Park, IL, USA). We measured blood glucose using the hexokinase method and standard equipment and methods, and the sex hormones (include testosterone, follicle stimulating hormone; luteinizing hormone; progestogen; estrogen, prolactin) and insulin using chemiluminescence method and its special kit. The iFlash 3000 Chemiluminescent Immunoassay Analyzer was used for the determination of AMH and INHB. Its reagents are AMH, INHB assay kits and the assay is a magnetic particle acridine ester chemiluminescence method.

#### Diagnostic criteria

The diagnostic criteria for PCOS in this study were selected from Chinese guidelines for the diagnosis and management of polycystic ovary syndrome (2018) [29]. Diagnostic criteria for PCOS: (1) sporadic ovulation or anovulation; (2) clinical manifestations of elevated androgen levels or hyperandrogenemia; (3) polycystic ovarian changes; (4) two of the above three criteria are met, and other causes of elevated androgen levels are excluded. Oligomenorrhea is defined as a menstrual cycle of more than 35 days (usually less than 8 cycles per year), which is a sign of anovulatory cycle [30]. Menorrhagia for more than 3 previous menstrual cycles or ≥ 6 months diagnosed as amenorrhea.

### Statistical analysis

SPSS 21.0 (IBM, Chicago, IL, USA) was used for statistical analysis. MedCalc 20.0.1 software for the production of hierarchical analysis charts. Data are presented as mean, standard variants and its 95% confidence intervals (95% CIs), the χ^2^-test was used to determine differences in the prevalence rates of the categorical variables between the three groups. One-way analyses of variance (ANOVA) and trend tests were used to compare the differences in each variable between the three groups. Furthermore, we conducted stratified analyses of ANOVA and trend tests by age, parity, AFC, and sex hormones. Spearman correlation analysis was used for correlation analysis between HOAM-IR, QUICKI, and ISI (gly).

## Results

### Baseline characteristics for patients with PCOS and healthy people

In this study, we have recruited 140 incident patients with PCOS who were aged 20–40 years between August 2019 and December 2021 at Shenzhen Baoan Mothers’and Children’s Hospital, Jinan University. The diagnoses of oligomenorrhea and amenorrhea of these were confirmed by follow-up until April 2022; while 64 patients with PCOS were clinically diagnosed as normal menstrual cycles after follow-up. The diagnosis of hyperandrogenemia was based on laboratory tests and clinical manifestations such as weight gain, hairy, acne, and IR [31]. The baseline clinical characteristics of the study population are shown in Table 1. There were no significant differences in general characteristics including ages and number of gestation and parturition, between patients with different menstrual manifestations of PCOS. However, we found that the levels of circulating insulin and HOMA-IR were statistically significant in patients with different menstrual manifestations (*P* values were 0.029 and 0.011, respectively). In the amenorrhea group, 58% of patients had a HOMA-IR index greater than 1.91, suggesting an association between amenorrhea and IR in patients with PCOS.

**Table 1:** Demographic characteristics and laboratory tests in 140 patients with PCOS included in this study

Parameters (%)	normal menstruation	oligomenorrhea	amenorrhea	*P*-value
Numbers	64	43	33	
Age (years)				0.978
≤ 25	37(58%)	24(56%)	19(58%)	
> 25	27(42%)	19(44%)	14(42%)	
Gestation				0.458
0	55(86%)	35(81%)	25(76%)	
> 0	9(14%)	8(19%)	8(24%)	
Parturition				0.410
0	59(92%)	40(93%)	28(85%)	
> 0	5(8%)	3(7%)	5(15%)	
BMI				< 0.001
≤ 27	32(50%)	2(5%)	29(88%)	
> 27	32(50%)	41(95%)	4(12%)	
Testosterone (ng/ml)				0.245
≤ 0.51	36(56%)	24(56%)	13(39%)	
> 0.51	28(44%)	19(44%)	20(61%)	
LH (mIu/ml)				0.845
≤ 10	35(55%)	25(58%)	17(52%)	
> 10	29(45%)	18(42%)	16(48%)	
FSH (mIu/ml)				0.783
≤ 5	34(53%)	20(47%)	16(48%)	
> 5	30(47%)	23(53%)	17(52%)	
LH/FSH				0.252
≤ 2	39(61%)	27(63%)	15(46%)	
> 2	25(39%)	16(37%)	18(54%)	
INHB (pg/ml)				0.036
≤ 74	29(45%)	24(56%)	24(73%)	
> 74	35(55%)	19(44%)	9(27%)	
AMH (ng/ml)				0.183
≤ 7.80	36(56%)	31(72%)	18(56%)	
> 7.80	28(44%)	12(28%)	15(44%)	
Progestogen (ng/ml)				0.814
≤ 0.73	57(89%)	37(86%)	28(85%)	
> 0.73	7(11%)	6(14%)	5(15%)	
E2(ng/l)				0.103
≤ 53	45(70%)	28(65%)	26(79%)	
> 53	9(30%)	15(35%)	7(21%)	
PRL (ng/ml)				0.935
≤ 19	38(59%)	24(56%)	19(58%)	
> 19	26(41%)	19(44%)	14(42%)	
Acne, Hairy				0.128
Yes	3(5%)	7(11%)	3(9%)	
No	61(95%)	36(89%)	30(91%)	
Glucose (mmol/l)				0.065
≤ 5.1	46(72%)	23(53%)	17(52%)	
> 5.1	18(28%)	20(47%)	16(48%)	
Insulin (mIu/l)				0.029
≤ 8.3	48(75%)	26(60%)	16(48%)	
> 8.3	16(25%)	17(40%)	17(52%)	
HOMA-IR				0.011
≤ 1.91	47(73%)	26(60%)	14(42%)	
> 1.91	17(27%)	17(40%)	19(58%)	
QUICKI				0.029
≤ 0.67	31(48%)	27(63%)	25(76%)	
> 0.67	33(52%)	16(37%)	8(24%)	
ISI (gly)				0.077
≤ 0.51	28(44%)	25(58%)	22(67%)	
> 0.51	36(54%)	18(42%)	11(33%)	
AFC				0.817
≤ 25	39(61%)	26(60%)	18(55%)	
> 25	25(39%)	17(40%)	15(45%)	
Hyperandrogenism				
Yes	64(100%)	26(60%)	20(61%)	< 0.001
No	0(0%)	17(40%)	13(39%)	

*Abbreviations*: *BMI* body mass index, *FSH* follicle stimulating hormone, *LH* luteinizing hormone, *E2* estrogen, *PRL* prolactin, *AMH* anti-Müllerian hormone, *INHB* inhibin B, *HOMA-IR* homeostasis model assessment: insulin resistance, *AFC* Antral follicle count, *QUICKI* quantitative insulin sensitivity check index, *ISI* insulin sensitivity indices. Notes: 0 and 1 for Gestation and Parturition mean yes or no, respectively. The cut-off values for age, BMI, sex hormones, AMH, INHB, glucose, insulin, HOMA-IR, QUICKI, ISI and AFC were the mean values of all patients

We also found a correlation between IR and other characteristics of patients with PCOS. When we grouped the patients by HOMA-IR levels, both INHB and progesterone levels were differently distributed between the two groups (*P* = 0.040 and 0.029, respectively) (Additional file 1: Table S1).

Additionally, there is a close association between the IR index QUICKI and the menstrual cycle (Table 2), so we performed Spearman correlation analyses between HOMA-IR and other IR indices and found that HOMA-IR was significantly correlated with QUICKI and ISI (gly) (both *P* values < 0.001) (Additional file 2: Table S2).

**Table 2:** Characteristics of indicators of IR, QUICKI and ISI (gly) in PCOS patients with different menstrual cycle performance (mean, 95% CI)

	normal menstruation	oligomenorrhea	amenorrhea	*P*	*P*_for trend_	*AdjP*
n	64	43	33			
HOMA-IR	1.610(1.370–1.850)	2.024(1.610–2.440)	2.352(1.960–2.750)	0.008	0.003	0.004
QUICKI	0.696(0.661–0.731)	0.664(0.622–0.706)	0.608(0.578–0.638)	0.007	0.002	0.037
ISI (gly)	0.552(0.497–0.607)	0.507(0.436–0.577)	0.448(0.375–0.520)	0.086	0.028	0.193

*Abbreviations*: *HOMA-IR* homeostasis model assessment: insulin resistance, *QUICKI* quantitative insulin sensitivity check index, *ISI* insulin sensitivity indices. Adjusted for age, *BMI* and INHB

### Association between insulin resistance and abnormal menstrual cycle in patients with PCOS

**Fig. 1:**
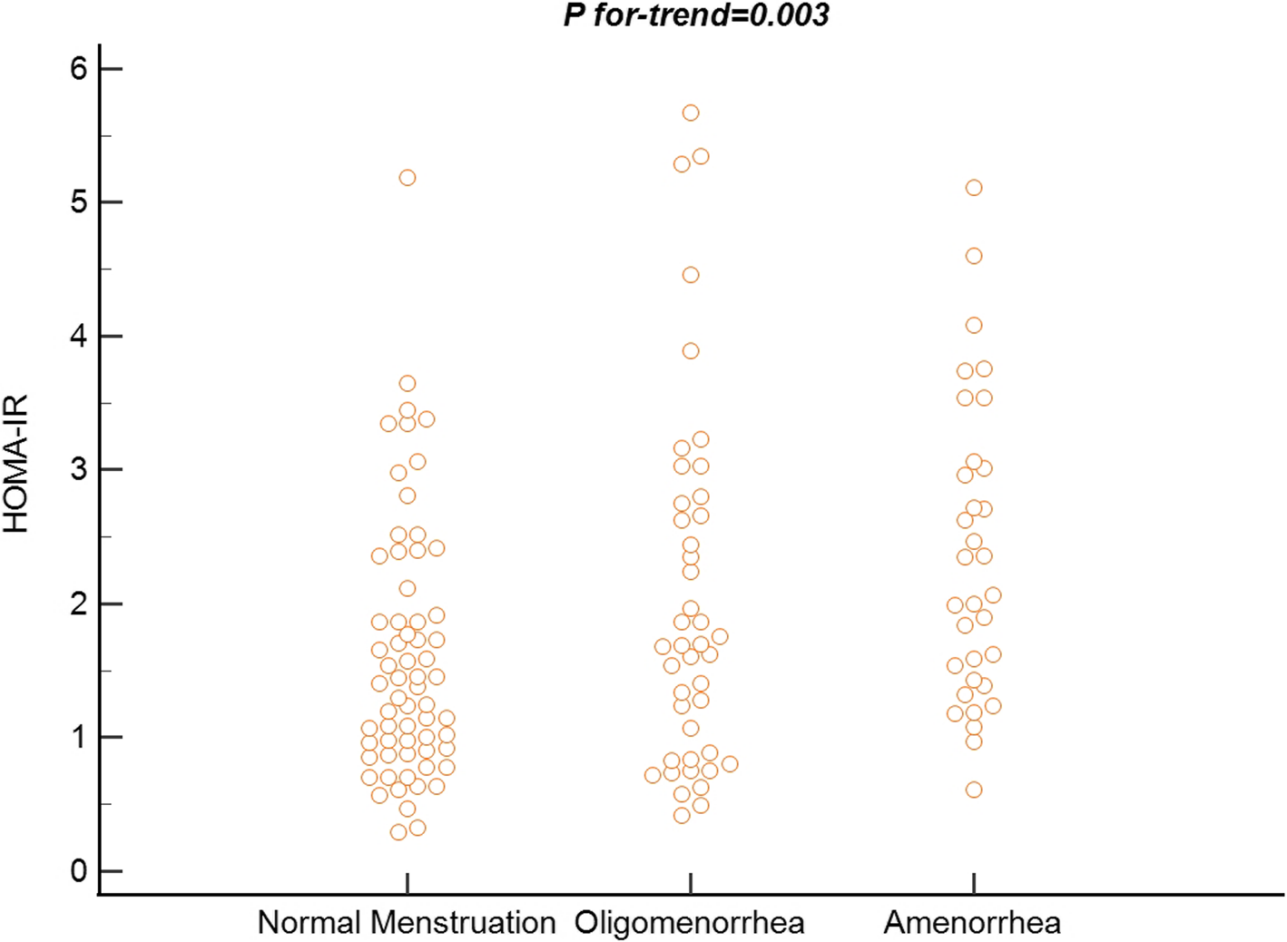
Association of insulin resistance and abnormal menstrual cycle in patients with polycystic ovary syndrome

As shown in Table 2, we found that compared to patients with normal menstruation, those with oligomenorrhea had a higher HOMA-IR index (mean: 2.020 [95%CI: 1.61–2.44] vs. 1.610 [95%CI: 1.37–1.85], *P* < 0.01), and those with amenorrhea had a further increased HOMA-IR index (mean: 2.350,95%CI: 1.96–2.75,* P* < 0.01). We also found a dose–response relationship between the increased HOMA-IR index and the level of menstrual cycle disorders (*P* for trend = 0.003, Fig. 1). Additionally, there was a significant difference between QUICKI levels and menstrual cycles (*P* = 0.007). The difference between ISI (gly) and different menstrual cycles was not statistically significant (*P* = 0.086). After adjustment for BMI, Age and INHB, HOMA-IR and QUICKI findings remained consistent, but ISI (gly) was no longer significantly associated with menstrual cycles (Table 2). These results might be due to the different sensitivities and related confounding factors of these three IR indices [26–28].

**Fig. 2:**
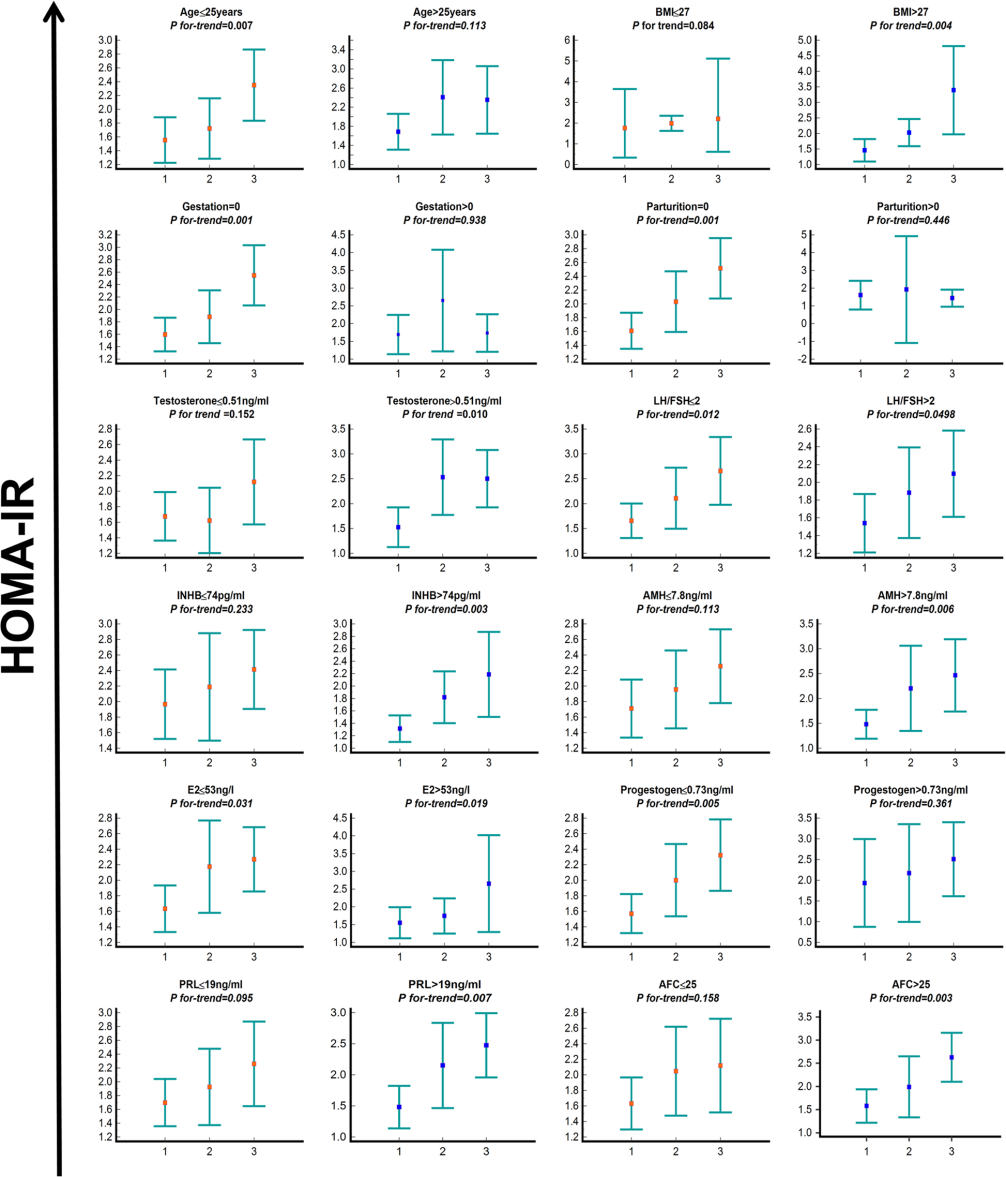
Relationship between HOMA-IR by Menstrual history in groups stratified by selected variables. Notes: 1 menstrual cycles, 2 oligomenorrhea, 3 amenorrhea

Although QUICKI as an indicator of IR was also associated with abnormal menstrual cycles (Table 2), it was clear that there was a strong correlation between HOMA-IR and QUICKI (*r* = -0.999) (Additional file 2: Table S2). Furthermore, the relationship between HOMA-IR and menstrualcycle disorders in patients with PCOS was stronger after adjusting for parameters compared to QUICKI (Table 2). Therefore, we selected HOMA-IR as an indicator of IR for further analyses. Among patients with different clinical presentations of the menstrual cycle, HOMA-IR indexes were significantly higher in the amenorrheic patients than in the oligomenorrhea and normal menstruation groups. This difference persisted when stratified by a range of demographics and various biochemical indicators (Fig. 2). Further stratification analyses showed that this dose–response relationship was more obvious in the patients who were younger than 25 years (*F* = 7.631, *P* for trend = 0.007), had no pregnancy history (*F* = 12.465,* P* for trend = 0.001), no parity history (*F* = 12.118,* P* for trend = 0.001), and higher levels of BMI (> 27, *F* = 8.961,* P* for trend = 0.004), testosterone (> 0.51 ng/ml, *F* = 6.967, *P* for trend = 0.010), AMH (> 7.8 ng/ml,* F* = 8.310,* P* for trend = 0.006), INHB (> 74.0 pg/ml, *F* = 9.928,* P* for trend = 0.003), prolactin (> 19 ng/ml, *F* = 7.776, *P* for trend = 0.007) and AFC (> 25, *F* = 4.759,* P* for trend = 0.003) (Fig. 2), and had a lower progesterone level (≤ 0.73 ng/ml,* F* = 8.026,* P* for trend = 0.005).

## Discussion

This study clarified the association between IR and amenorrhea in patients with PCOS. It was evident that when we grouped the samples by the menstrual cycle, the level of IR in amenorrheic patients was significantly higher than in patients with sparse and normal menstruation. Moreover, when we stratified the analysis by general characteristics and hormone-related indicators, the association between IR and abnormal menstrual cycles was more significant in the patients with PCOS who were younger than 25 years, had not gestations and no parity, had an elevated AFC, and had higher levels of AMH, INHB, testosterone and prolactin levels.

Although a correlation between abnormal menstrual cycles and IR has been previously demonstrated in patients with PCOS [16, 23], this study focuses on the dose–response relationship between IR levels and menstrual cycle disorders. Dose–response relationship can quantitatively show the strength of association. The “Dose” does not only represent drugs, but also many types of exposure [17–19]. Dose–response relationship is widely used for metabolic indexes [20, 25], but has not been used for IR in patients with PCOS. Our study established a dose–response relationship between IR and abnormal menstrual cycles. IR is very harmful for women of reproductive age, and it is associated with infertility and comorbidity such as type 2 diabetes. Therefore, early diagnosis and intervention are of great importance for patients with PCOS.

In stratified analyses, we found that the relationship between IR and abnormal menstrual cycles was affected by many environmental factors. For example, this association was more significant in the PCOS with no gestational and no delivery history. Our results are consistent with the fact that IR is associated with infertile in the patients with PCOS [4, 5], who usually have oligomenorrhea and amenorrhea.

BMI is another confounding index, which is close related to IR. The stratified analyses showed that the dose–response relationship between IR and irregular menstrual cycle was significant in the patients with higher BMI (> 27 kg/m^2^). On the contrary, patients with lower BMI level have less chance to expose to oligomenorrhea and amenorrhea. For example, half of patients with normal menstrual cycles have a lower BMI (≤ 27 kg/m^2^) in this study. Consistent with the correlation between BMI and IR [32], 73% of these patients had a lower HOMA-IR level (≤ 1.91). Consequently, those with lower BMI will have less chances to expose to excessive hormones and progressive reduction in the frequency of menstrual cycles, because of lower HOMA-IR levels [33–35]. Therefore, lifestyle (including dietary habits, physical activity, and/or behavior change) management has been the first line of therapy for weight gain in PCOS [36]. Oral medications (metformin, orlistat, liraglutide) and surgical weight loss have a positive effect on improving IR, hyperandrogenemia, menstruation and pregnancy outcomes in patients with PCOS [36–39]. However, due to the adverse and side effects of oral medication, the limitations of daily lifestyle changes, there are other ways to improve IR. Some studies have shown that acupuncture can improve IR-related metabolic disorders (including hyperandrogenemia, glucose and overweight), and perhaps future clinical work could include acupuncture for treating PCOS [40].

The stratified analyses also showed that the association between IR and abnormal menstrual cycles was more significant in the patients aged less than 25 years than in older patients. Young patients with PCOS have significantly higher levels of IR than older ones, and are at significantly higher risk of anxiety and depression [41], which can affect menstrual cycles. A case–control study showed that young patients with PCOS had significantly higher granzyme-B levels and an increased risk of IR [42], which can affect sex hormone levels, thus a risk of abnormal menstrual cycles. Therefore, younger patients with PCOS have higher levels of IR and are in greater need of early diagnosis and intervention of oligomenorrhea and amenorrhea.

Our results showed that the association between IR and abnormal menstrual cycles was more evident in the patients with high AFC number as well. As for AFC, there are no uniform diagnostic criteria for PCOS, the most widely used being the Rotterdam criteria [43]. In the Rotterdam criteria, the diagnosis of polycystic ovaries is 12 or more luminal follicles of 2–9 mm in diameter or an increase in ovarian volume > 10 cm^3^ [44]. Hyperinsulinemia caused by IR increases androgen production through a mitogenic effect on ovarian follicular membrane cells, which can cause interstitial hyperplasia, and there is evidence of a positive correlation between IR and ovarian volume, but no clear association between IR and AFC [45]. In contrast, the results of this study demonstrate an association between IR levels and amenorrhea that is more evident in patients with AFC number > 25. Larger population studies are warranted to clarify the relationship between IR and AFC. More attention should be paid to prevent amenorrhea in patients with PCOS with both IR and higher AFC number.

We also found that the association between IR and abnormal menstrual cycles is affected by many hormones, such as AMH, INHB, progesterone, testosterone, and prolactin. AMH is a member of the transforming growth factor beta (TGF-β) family, which is secreted by the anterior and small antral follicles [11]. It increases significantly in patients with PCOS and correlates with the severity of the disease, including oligomenorrhoea, and amenorrhea [46, 47]. Some studies considered that the level of AMH was not significantly correlated with IR [48]; while other studies have shown that AMH level was negatively correlated with HOMA-IR index [14, 15]. The association between AMH and IR does not have a clear description and needs to be elucidated by further studies. INHB is a hormone secreted by the granulosa cells of the antral follicle and is a member of the TGF-β family, like AMH [49]. It stimulates follicular membrane cell androgen production, and is elevated in patients with PCOS [50]. Many factors influenced the secretion of INHB, such as FSH and insulin-like growth factor. However, few studies have shown the relationship between INHB and IR [14, 15], which need to be further studied and explored. In this study we found that IR is significantly associated with INHB and progesterone (*P* = 0.040 and 0.029, respectively). The latter hormone is usually increased in the patients with diabetes or gestational diabetes, suggesting its critical role in IR [51]. As for testosterone, it is usually increased in patients with PCOS, especially in those with amenorrhea [4, 5]. Multiple studies have demonstrated a strong correlation between elevated androgen levels and IR [10, 13], via the activation of the pituitary–gonadal axis and the ACTH/SHBG signals. The increased level in testosterone is independently associated with a significant decrease in SHBG, which further makes IR more severe [52, 53]. Prolactin is a hormone that is closely related to metabolism. Prolactin levels are usually higher in the patients with PCOS, and hyperprolactinemia may be associated with anovulation [54]. Some studies have demonstrated a strong association between higher prolactin levels and IR [55], and a negatively association with LH and LH/FSH in PCOS [56]. The association between serum prolactin level and IR are also seen in the patients with obese and diabetes [57, 58]. Taken together with above reports, our results of the effects of these hormones on the association between IR and abnormal menstrual cycles are functionally plausible. We argue that higher levels of HOMA-IR and aberrant hormone profiles are biomarkers of infertility in patients of child-bearing age with PCOS.

In addition to irregular menstrual cycles, the decrease in oocytes and endometrial dysfunction caused by various hormonal metabolism disorders, such as IR, further lead to infertility or poor pregnancy outcomes in patients with PCOS [59, 60]. Therefore, the early detection and diagnosis of IR is of great value for the preservation of fertility in patients with PCOS.

This study also has some limitations in that we selected a population with a small sample size, which affected the final results. Furthermore, the sample was selected from local patients in Shenzhen, a southern Chinese city, which is somewhat geographically diverse and unrepresentative of the clinical presentation and examination findings of most patients with PCOS.

## Conclusions

Our study has established dose–response relationship between IR and abnormal menstrual cycles in the patients with PCOS, which can be affected by age, BMI, AFC and aberrant hormone levels. In clinical work, due to the problem of timing of diagnosis in amenorrheic patients, we may be able to combine endocrine and metabolic indicators to intervene earlier and improve the prognosis of PCOS.

## Supplementary Information

**Additional file 1: Table S1.** Relationship between IR and other clinical phenotypes in patients with PCOS.

**Additional file 2: Table S2.** Spearman rank correlations between IR indices.

## Data Availability

The data sets used and/or analyzed during the current study are available from the corresponding author on reasonable request.

## Abbreviations

BMI: Body mass index
FSH: Follicle stimulating hormone
LH: Luteinizing hormone
E2: Estrogen PRL, prolactin
AMH: Anti-Müllerian hormone
INHB: Inhibin B
HOMA-IR: Homeostasis model assessment: insulin resistance
AFC: Antral follicle count
QUICKI: Quantitative insulin sensitivity check index
ISI: Insulin sensitivity indices
