# Supplementary material for: Association between insulin resistance and abnormal menstrual cycle in Chinese patients with polycystic ovary syndrome
Source: J Ovarian Res. 2023 Feb 23;16:45. doi: 10.1186/s13048-023-01122-4 (PMC9948335; doi:10.1186/s13048-023-01122-4)
Supplement: Supplementary file 1 — Additional file 1: Table S1. Relationship between IR and other clinical phenotypes in patients with PCOS. [file 13048_2023_1122_MOESM1_ESM.docx]

Appendix 1: Table S1 Relationship between IR and other clinical phenotypes in patients with PCOS

| Parameters (%) | HOMA-IR≤1.91 | HOMA-IR>1.91 | χ^2^-value | *P*-value |
| --- | --- | --- | --- | --- |
| Numbers | 87 | 53 |  |  |
| Age (years) |  |  | 2.277 | 0.131 |
| ≤25 | 54(62%) | 26(49%) |  |  |
| >25 | 33(38%) | 27(51%) |  |  |
| Gestation |  |  | 0.059 | 0.807 |
| 0 | 72(83%) | 43(81%) |  |  |
| >0 | 15(17%) | 10(19 %) |  |  |
| Parturition |  |  | 0.306 | 0.580 |
| 0 | 78(90%) | 49(92%) |  |  |
| >0 | 9(10%) | 4(8%) |  |  |
| BMI |  |  | 1.217 | 0.270 |
| ≤27 | 36(41%) | 27(51%) |  |  |
| >27 | 51(59%) | 26(49%) |  |  |
| Testosterone (ng/ml) |  |  | 0.845 | 0.358 |
| ≤0.51 | 48(55%) | 25(47%) |  |  |
| >0.51 | 39(45%) | 28(53%) |  |  |
| LH/FSH |  |  | 0.222 | 0.637 |
| ≤2 | 49(56%) | 32(60%) |  |  |
| >2 | 38(44%) | 21(40%) |  |  |
| INHB (pg/ml) |  |  | 4.198 | 0.040 |
| ≤74 | 42(48%) | 35(66%) |  |  |
| >74 | 45(52%) | 18 (34%) |  |  |
| AMH (ng/ml) |  |  | 0.004 | 0.949 |
| ≤7.80 | 53(61%) | 32(60%) |  |  |
| >7.80 | 34(39%) | 21(40%) |  |  |
| Progestogen (ng/ml) |  |  | 4.748 | 0.029 |
| ≤0.73 | 80(92%） | 42(79%) |  |  |
| >0.73 | 7(8%) | 11( 21%） |  |  |
| E2(ng/L) |  |  | 0.339 | 0.560 |
| ≤53 | 60(69%) | 39(74%) |  |  |
| >53 | 27(31%) | 14(26%) |  |  |
| PRL (ng/ml) |  |  | 0.884 | 0.347 |
| ≤19 | 53(61%) | 28(53%) |  |  |
| >19 | 34(39%) | 25(47%) |  |  |
| AFC |  |  | 2.459 | 0.117 |
| ≤25 | 56(64%) | 27(51%) |  |  |
| >25 | 31(36%) | 26(49%) |  |  |
| Hyperandrogenism |  |  | 0.023 | 0.879 |
| Yes | 68(78%) | 42(79%) |  |  |
| No | 19(22%) | 11(21%) |  |  |
