# Supplementary material for: Association between insulin resistance and abnormal menstrual cycle in Chinese patients with polycystic ovary syndrome
Source: J Ovarian Res. 2023 Feb 23;16:45. doi: 10.1186/s13048-023-01122-4 (PMC9948335; doi:10.1186/s13048-023-01122-4)
Supplement: Supplementary file 2 — Additional file 2: Table S2. Spearman rank correlations between IR indices. [file 13048_2023_1122_MOESM2_ESM.docx]

**Appendix 2: Table S2.** Spearman rank correlations between insulin resistance indices.

|  | | ISI (gly) | QUICKI | HOMA-IR | *P* |
| --- | --- | --- | --- | --- | --- |
| HOMA-IR | -0.575 | | -0.999 |  | <0.001 |
| QUICKI | 0.576 | |  | -0.999 | <0.001 |
| ISI (gly) |  | | 0.576 | -0.575 | <0.001 |
